# MARes-Net: multi-scale attention residual network for jaw cyst image segmentation

**DOI:** 10.3389/fbioe.2024.1454728

**Published:** 2024-08-05

**Authors:** Xiaokang Ding, Xiaoliang Jiang, Huixia Zheng, Hualuo Shi, Ban Wang, Sixian Chan

**Affiliations:** ^1^ College of Mechanical Engineering, Quzhou University, Quzhou, China; ^2^ Department of Stomatology, Quzhou People’s Hospital, The Quzhou Affiliated Hospital of Wenzhou Medical University, Quzhou, China; ^3^ School of Mechanical Engineering, Hangzhou Dianzi University, Hangzhou, China; ^4^ College of Computer Science and Technology, Zhejiang University of Technology, Hangzhou, China

**Keywords:** jaw cyst, residual connection, U-Net, scale-aware feature extraction, multi-scale compression excitation, attention gate

## Abstract

Jaw cyst is a fluid-containing cystic lesion that can occur in any part of the jaw and cause facial swelling, dental lesions, jaw fractures, and other associated issues. Due to the diversity and complexity of jaw images, existing deep-learning methods still have challenges in segmentation. To this end, we propose MARes-Net, an innovative multi-scale attentional residual network architecture. Firstly, the residual connection is used to optimize the encoder-decoder process, which effectively solves the gradient disappearance problem and improves the training efficiency and optimization ability. Secondly, the scale-aware feature extraction module (SFEM) significantly enhances the network’s perceptual abilities by extending its receptive field across various scales, spaces, and channel dimensions. Thirdly, the multi-scale compression excitation module (MCEM) compresses and excites the feature map, and combines it with contextual information to obtain better model performance capabilities. Furthermore, the introduction of the attention gate module marks a significant advancement in refining the feature map output. Finally, rigorous experimentation conducted on the original jaw cyst dataset provided by Quzhou People’s Hospital to verify the validity of MARes-Net architecture. The experimental data showed that precision, recall, IoU and F1-score of MARes-Net reached 93.84%, 93.70%, 86.17%, and 93.21%, respectively. Compared with existing models, our MARes-Net shows its unparalleled capabilities in accurately delineating and localizing anatomical structures in the jaw cyst image segmentation.

## 1 Introduction

Jaw cyst is a cystic mass that develops from dental tissue, dental epithelium, or residual epithelial cells, which is usually filled with fluid. Its causes vary from person to person, but usually involve an abnormal disruption in the development of tooth structures, leading to the growth of cystic lesions. Diagnosing a jaw cyst often requires multiple methods. Initially, clinicians perform visual examinations and palpations, relying on their extensive experience and expertise to detect the presence and general location of the lesion. CT scans and X-rays are then used to provide detailed images of the internal structure, helping to confirm the size, shape, and relationship of the cyst to surrounding bones and teeth. Treatment for jaw cysts usually depends on the size, type, and location of the cyst and may include surgery or medication. Early diagnosis and intervention are crucial to prevent further cyst development and to reduce patient discomfort. Visual examination and palpation rely heavily on the physician’s clinical experience and expertise. However, the size and shape of jaw cysts can change over time, and the complexity of surrounding anatomy can interfere with accurate judgment. While CT scans and X-rays offer more detailed image analysis, they are also influenced by the evolving nature of the cyst and the complexity of nearby structures. Although these methods are widely used in clinical practice, each has limitations. Visual examinations and palpations depend on the physician’s skill and the patient’s clear communication, whereas imaging methods may be constrained by changes in cyst characteristics and the complexity of the surrounding anatomy.

As an important research direction of artificial intelligence, deep-learning becomes a promising solution to overcome the above challenges. By utilizing neural networks and large data sets, deep-learning algorithms can autonomously learn patterns and features from medical images to provide a more objective and accurate diagnosis. With the continuous development and maturity of deep-learning, its impact on clinical practice will be further expanded and large number of algorithms are applied to medical image segmentation (Zhao et al., 2024; Mikhailov et al., 2024; Zhang et al., 2023; Peng et al., 2023; Buddenkotte et al., 2023). For example, U-Net (Ronneberger et al., 2015) is a deep learning architecture that has many advantages due to its unique U-shaped network and skip connection mechanism. Firstly, the U-shaped structure allows information transfer between encoders and decoders to help extract multi-level features and preserve high-resolution spatial information, which can better capture target details and context information. Secondly, the skip connection (Nodirov et al., 2022; Shi et al., 2019; Wang et al., 2023) mechanism enables the decoder to effectively utilize the feature maps in the encoder, thereby improving the quality of segmentation results. In addition, U-Net are usually combined with data augmentation techniques to achieve good performance with a small amount of labeled data. Based on the above advantages, many scholars conducted in-depth research based on U-Net, and achieved extremely obvious improvements. Among them, He et al. (2016) proposed a residual learning framework, which aimed to simplify the complexity of training deeper networks. By combining residual connections and U-Net structure, ResUnet can train deep networks more efficiently and achieve better performance in image segmentation tasks. Yu et al. (2022) proposed a two-branch network for region segmentation of jaw cysts and tumors. Under this dual-path structure, the model can effectively capture both global contextual information and local fine-grained features within the image data. Furthermore, the segmentation subnetwork embedded within this framework serves as a powerful tool for refining classification performance and facilitating the interpretation of diagnostic results. Kanauchi et al. (2023) proposed a new method combining YoLov5 and UNet++ to address the challenge of renal cyst detection in ultrasound imaging. By inputting ultrasound images of renal cysts into YoLov5 and then seamlessly integrated into the UNet++ framework, it can predict the location of lesions with high accuracy in a very short time and provide doctors with a fast and reliable diagnostic tool. Rai and Chatterjee (Rai and Chatterjee, 2021) proposed LeU-Net architecture inspired by the renowned Le-Net and U-Net. Drawing upon the strengths of these frameworks, LeU-Net strikes a delicate balance between model complexity and computational efficiency, positioning it as a versatile tool for image classification tasks.

However, despite deep-learning has achieved significant successes, several formidable challenges remain. Firstly, medical images are usually limited in quantity, and the labeling process is not only time-consuming but also labor-intensive, which severely limits the training of deep-learning models. Secondly, medical images are particularly susceptible to various forms of noise, artifacts, and even deliberate manipulation, which can significantly compromise the performance and robustness of deep learning models. Furthermore, an additional challenge stems from the inherent class imbalance present in medical image datasets. Lastly, the intricate nature of medical images, characterized by complex anatomical structures and overlapping features, poses a significant obstacle to accurate segmentation. Despite advances in deep-learning techniques, existing medical image segmentation algorithms are still unable to achieve consistent and reliable clinical results.

In the domain of medical image analysis, due to the fuzziness and uncertainty of jaw cyst images, traditional deep-learning performs poorly in global information modeling and multi-scale feature extraction, and with the deepening of the network, problems such as gradient disappearance will occur, leading to the deterioration of segmentation performance. In response to the above problems, we established a MARes-Net framework based on residual network for jaw cyst segmentation. The MARes-Net framework utilizes a residual network architecture to mitigate the disappearing gradient problem and facilitate deeper network training. On this basis, several innovative modules are integrated: including scale-aware feature extraction module, multi-scale compression excitation, attention gate module. Through the synergistic fusion of these modules, the segmentation network demonstrates notable improvements in performance. Specifically, metrics such as precision, recall, IoU and F1-score have all shown significant improvements, with values reaching remarkable levels of 93.84%, 93.70%, 86.17%, and 93.21% on the original jaw cyst dataset. The contributions of this article have the following three points:

1) A scale-aware feature extraction module is proposed, which uses expanded convolution and CBAM to expand the receptive field and extract key feature information in channels and spatial dimensions.
2) A multi-scale compression excitation module is introduced to compress feature maps layer by layer so that the network can have richer contextual information.
3) An attention gate module has been introduced to selectively focus the network’s attention on salient regions within the image.

## 2 Materials and methods

In recent years, the advancement of deep-learning technology has heralded major breakthroughs in the field of medical image segmentation. Among various architectures, U-Net network has emerged as a formidable contender, which can offer clinicians and researchers a robust framework for clinical diagnosis. Our proposed MARes-Net builds upon the foundation of the U-Net architecture, which is mainly consisted of residual connection, down-sampling, multi-scale compression excitation module, scale-aware feature extraction module, up-sampling, attention gate module, and output layer. Specifically, firstly, the residual connection is introduced in the encoding and decoding stages of traditional convolutional blocks, so that the network can learn the residual function more efficiently. This enhancement enables the model to better adapt to different datasets and real-world scenarios, while minimizing the risk of overfitting. Moreover, the integration of multi-scale compression excitation module and scale-aware feature extraction module further enriches the feature representation capability of MARes-Net. The former can dynamically adjust the weight of feature mapping by compression excitation operation to strengthen the attention of salient features and enhance the generalization ability. Meanwhile, the scale-aware feature extraction module uses extended convolution technology to capture the feature representation of spatial information more comprehensively and promote more detailed segmentation results. Lastly, the attention-gate module enhances the network’s focus on key input data and dynamically adjusts the weights to prioritize salient features. This meticulous attention to the importance of input information significantly improves the model’s performance in specific tasks, with greater accuracy and robustness in a variety of medical imaging scenarios.

**FIGURE 1:**
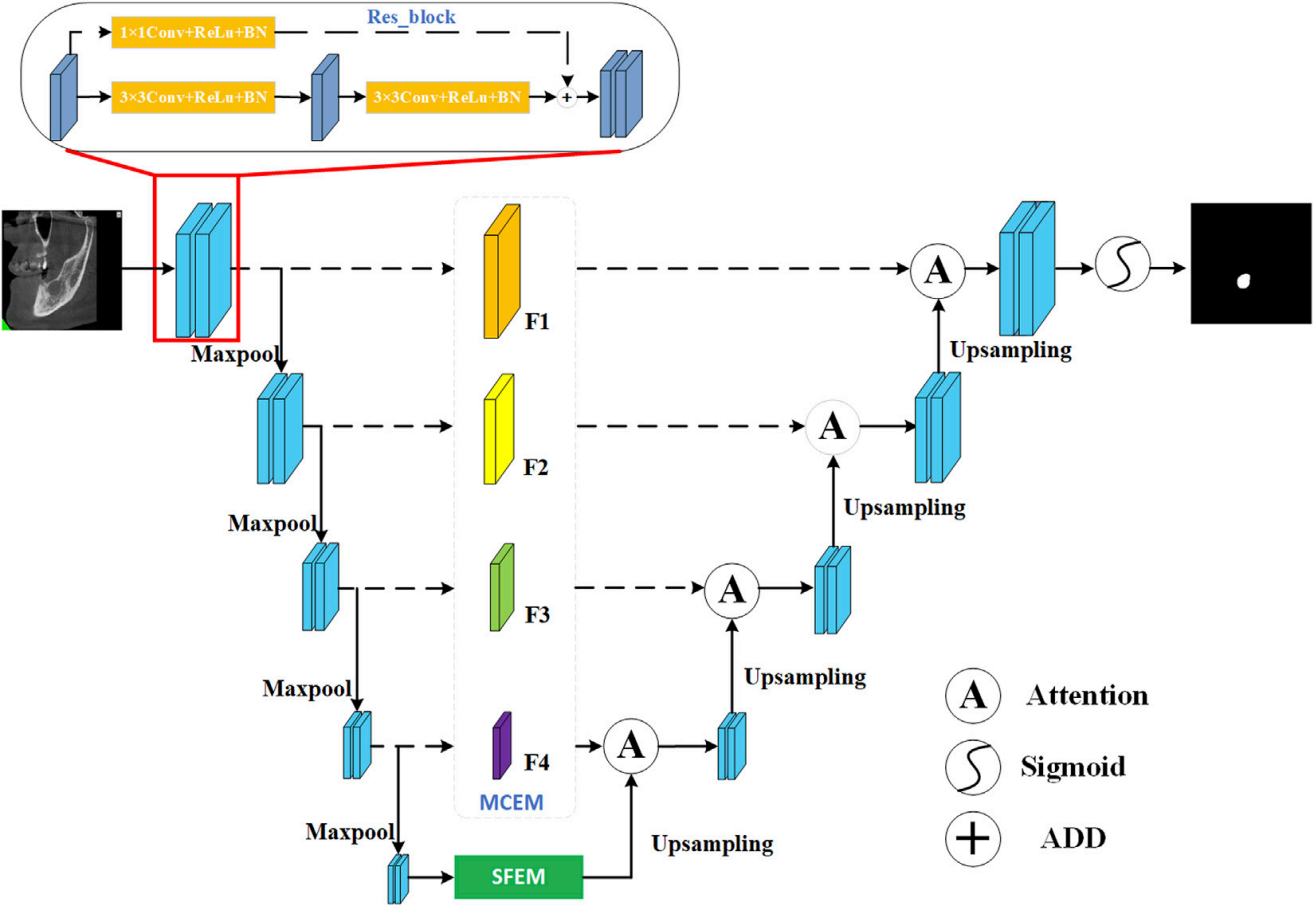
Network architecture of our MARes-Net.

Figure 1 is a visual representation of the MARes-Net architecture, which shows the interaction of the various components. Unlike the original U-Net and ResNet architectures, we have integrated the SFEM in the skip connections. This module effectively merges contextual information, allowing shallow and deep features to collaborate intelligently. At the network’s deepest level, we introduced the MCEM, which leverages dilated convolutions to delve deeper into the data and capture more significant features. Additionally, we incorporated attention mechanisms that dynamically adjust the spatial position of each pixel in the feature map, thereby refining the model’s focus. Finally, by utilizing the sigmoid function for lesion segmentation, we achieve precise and effective results. In subsequent chapters, we will delve into the above important modules and clarify their contribution to medical image analysis.

### 2.1 Residual connection

In traditional neural networks, the input of each layer undergoes a series of nonlinear transformations to gradually extract high-level features, which are then used as the input of the subsequent layer. However, as the network depth increases, the gradient gradually decreases during backpropagation. This phenomenon makes training exceptionally difficult and hinders the network’s ability to learn and adapt effectively. To overcome this problem, residual connection (Yoganathan et al., 2023; Chen et al., 2022; Liu et al., 2023) is introduced, which alleviate the vanishing gradient situation by directly funneling the output of one layer into the input of the next. This direct path promotes a smooth flow of gradients, which allows them to traverse shallower layers more easily. If the output of the previous layer is represented as x and the input of the subsequent layer is represented as y, the expression of the residual connection is:

$$y=f\left(x\right)+x$$ (1)

The addition of the identity mapping term x ensures the preservation of original information, while the function $f\left(x\right)$ introduces additional transformations tailored to capture intricate features and patterns. Consequently, this optimization design can optimize the network parameters more effectively and improve the training efficiency of deep neural networks.

### 2.2 Multi-scale compression excitation module

In U-Net, skip connections are widely used to build the encoder-decoder structure. These connections establish direct links between feature maps in the encoder and their corresponding counterparts in the decoder, which helps to retrieve details lost during down-sampling and improve segmentation performance. Furthermore, the squeeze-and-excitation (SE) module (Yu et al., 2022; Zhang et al., 2022; Chowdary et al., 2023) is an attention mechanism designed to enhance the performance of deep neural networks. It achieves this by introducing a learning process that dynamically adjusts the importance of each feature map channel, thereby improving the network’s performance on specific tasks. The core concept of the SE module is to use global information to calculate importance weights for each channel, which are then used to reweight the feature map. This approach not only significantly enhances the network’s performance in various computer vision tasks, such as image classification, object detection, and semantic segmentation, but also effectively improves feature expression and discrimination without adding additional computational complexity.

**FIGURE 2:**
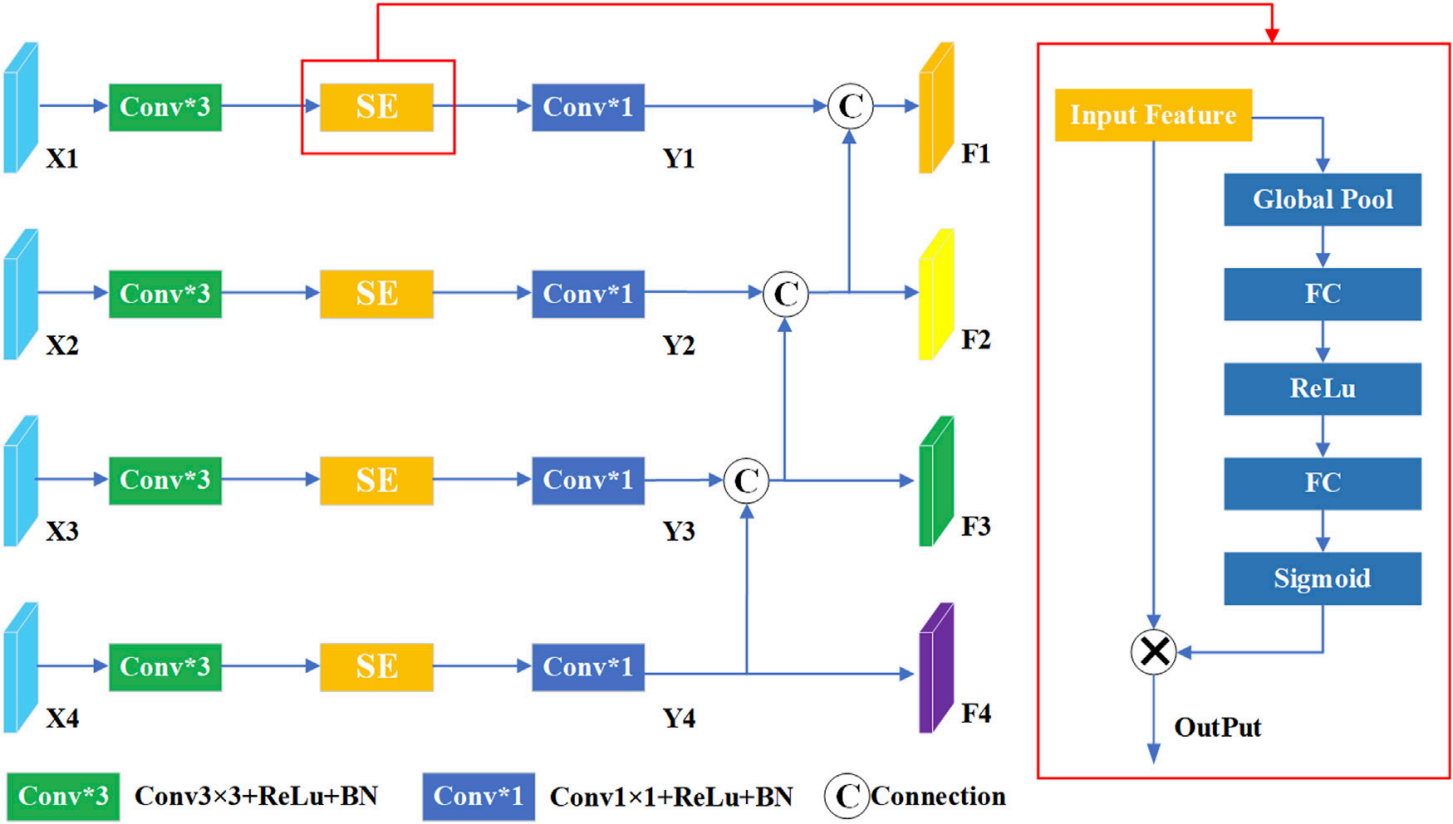
The structure of multi-scale compression excitation module.

To further elevate model performance, we introduce a multi-scale compressed excitation module, which accepts feature maps from different resolutions as input. As shown in Figure 2, the input feature mappings from layer one to layer four are represented as {X1, X2, X3, X4}. Initially, these features undergo convolution operations with a 3 × 3 convolution kernel, serving to extract and enhance key features within each layer. Following this, SE module is used to adaptively weight the feature mappings of each channel, and then further processed by 1 × 1 convolution layer, culminating in the derivation of new feature sets denoted as {Y1, Y2, Y3, Y4}. In this framework, the output features of layer four are further split into two different paths, one path continues uninterrupted towards the specified output F4, while the other diverges and intersects with the output Y3 of Layer three. This strategic divergence and merging mechanism imbue the features traversing the layer three path with contextual insights garnered from layer four, enriching their understanding and enhancing their discriminative power. Similarly, F1 and F2 are derived similarly to F3. Finally, the final output {F1, F2, F3, F4} is passed to the corresponding decoding layer respectively. In summation, the framework embodies a complex multi-scale compression excitation strategy, which can deal with feature mappings of different resolutions skillfully. By seamlessly integrating SE modules, the network gains the ability to adaptively allocate attention to the functions of different channels. In addition, despite the inherent information differences between shallow and deep features, their fusion produces a collaborative mix that ultimately enhances the model’s performance and ability to generalize across specialized tasks.

### 2.3 Scale-aware feature extraction module

**FIGURE 3:**
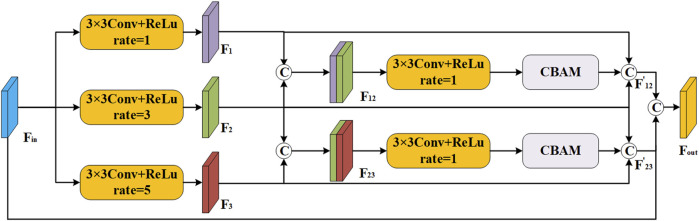
The structure of scale-aware feature extraction module.

To effectively capture the intricate detail present in multi-scale regions of interest, we build a scale-aware feature extraction module that combines atrous convolution (Yin et al., 2023; Ying et al., 2023) and convolutional block attention module (CBAM) (Nguyen and Nguyen, 2024; Xiong et al., 2024). As depicted in Figure 3, this module can increase the receptive domain of the convolution kernel by introducing additional intervals, and it can capture a larger range of contextual information without adding additional parameters. Specifically, we first split $F_{in}$ into three branches and use different expansion sizes (1, 3, 5) to capture a rich array of spatial hierarchies and contextual intricacies present. Then, we employ the element-wise operation as a pivotal step in our methodology, which is defined as:

$$\begin{array}{l} F_{12}=F_{conv}^{3\times 3,r=1}\left(F_{in}\right)⊕F_{conv}^{3\times 3,r=3}\left(F_{in}\right)\\ F_{23}=F_{conv}^{3\times 3,r=3}\left(F_{in}\right)⊕F_{conv}^{3\times 3,r=5}\left(F_{in}\right) \end{array}$$ (2)

where $F_{conv}^{n\times n,r}$ represents the convolution with kernel size denoted by n, and r represents the spacing rate of the atrous convolution, ⊕ represents the connection. Subsequently, 3 × 3 convolution is performed on feature maps F_12_ and F_23_, and then CBAM is used to lock more areas of interest to further enhance the model’s focus on key features. Finally, combining the above feature maps, the new functional map $F_{out}$ is:

$$\begin{array}{l} F_{12}^{\prime }=CBAM\left(F_{conv}^{3\times 3,r=1}\left(F_{12}\right)\right)⊕F_{1}⊕F_{2}\\ F_{23}^{\prime }=CBAM\left(F_{conv}^{3\times 3,r=1}\left(F_{23}\right)\right)⊕F_{2}⊕F_{3}\\ F_{out}=F_{12}^{\prime }⊕F_{23}^{\prime }⊕F_{in} \end{array}$$ (3)

**FIGURE 4:**
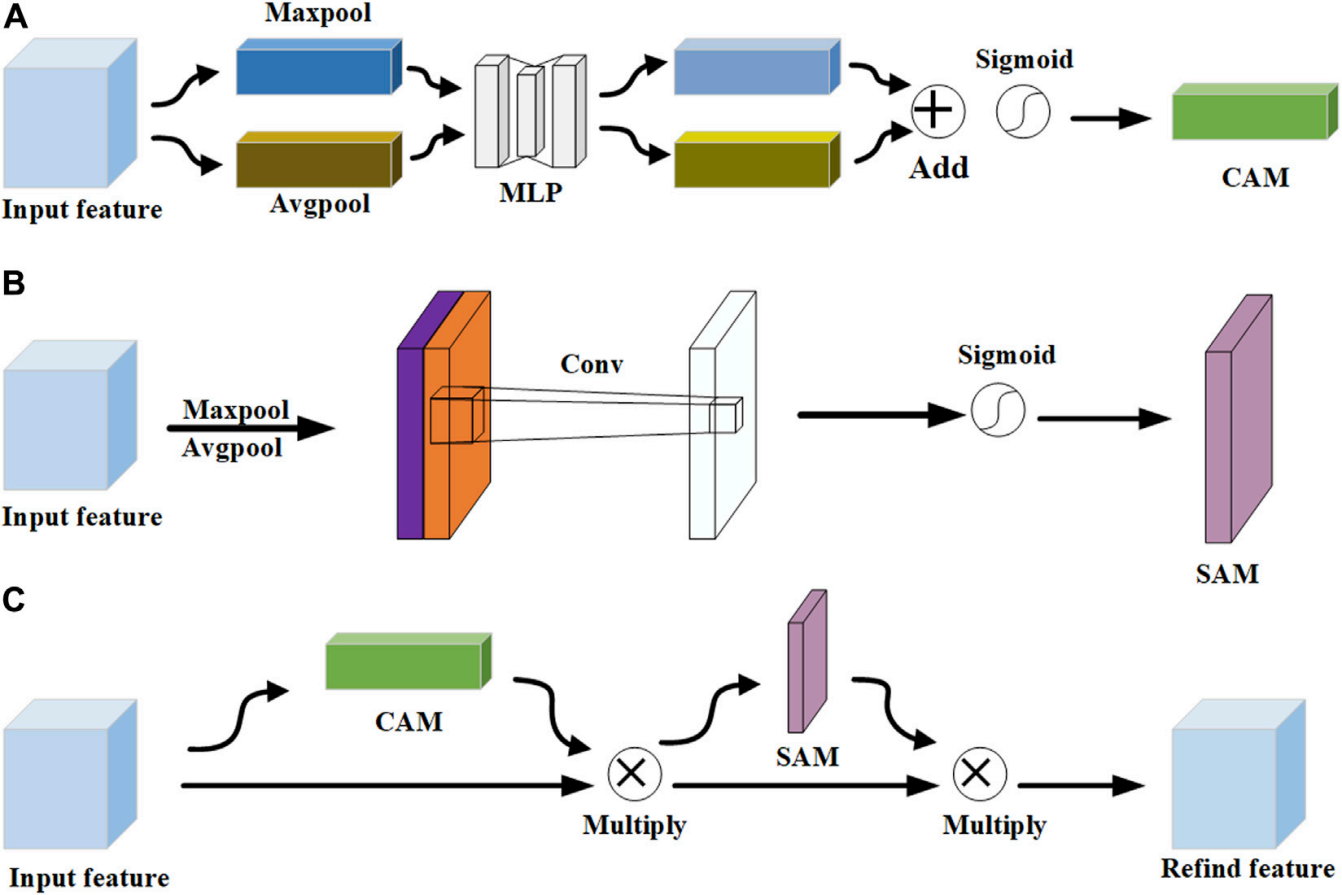
The structure of CBAM. **(A)** CAM. **(B)** SAM. **(C)** CBAM.

In the scale-aware feature extraction module, the CBAM represents a pivotal innovation in the realm of neural network architectures, particularly in the domain of computer vision. As depicted in Figure 4, it contains two sub-modules: channel attention module (CAM) (Lee et al., 2020; Song et al., 2023) and spatial attention module (SAM) (Tao et al., 2019; Zhang et al., 2023). The advantage of CBAM is that it cleverly integrates the two submodules of channel attention and spatial attention, which enables the network to identify and prioritize key channels and spatial regions in the input feature map. According to the structure of CAM in Figure 4A, the maximum and average pooling operations are applied to the feature map to generate two different 1 × 1 × C feature mappings. Subsequently, these feature maps undergo processing through a two-layer multi-layer perceptron (MLP) with shared weights for learning the dependencies between channels. Finally, the output of MLP undergoes an elemental summation, followed by weighting through the sigmoid function. The result of CAM is calculated as:

$$M_{CA}\left(F\right)=\sigma \left(MLP\left(AvgPool\left(F\right)\right)\right)⊕\sigma \left(MLP\left(MaxPool\left(F\right)\right)\right)$$ (4)

where $\sigma \left(\cdot \right)$ represents the sigmoid function and $M_{CA}\left(F\right)$ is the output of CAM.

According to the structure of SAM in Figure 4B, the maximum and average pooling operations are performed to extract different aspects of the spatial information. Subsequently, these two feature maps are combined by concatenating them along the channel dimension. This splicing operation merges the distinct information captured by the maximum and average pooling into a single, unified feature map. Then, a 7 × 7 convolution kernel is applied to perform channel dimensionality reduction on the spliced feature map, and the dimensionality is reduced to a single-channel feature map, that is, the size is H×W×1. Finally, the dependencies between spatial elements are learned through the sigmoid function to generate the weights of the spatial dimensions. The result of SAM is calculated:

$$M_{SA}\left(F\right)=\sigma \left(F_{conv}^{7\times 7}\left(\left[AvgPool\left(F\right);MaxPool\left(F\right)\right]\right)\right)$$ (5)

### 2.4 Attention gate module

**FIGURE 5:**
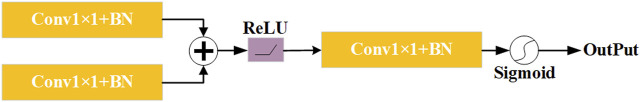
The structure of attention gate module.

The attention gate module (Hao and Li, 2023; Chen et al., 2024) is an advanced mechanism designed to significantly enhance the performance of neural networks, particularly in complex tasks such as image segmentation. This module plays a crucial role in refining the model’s focus by dynamically adjusting the importance of each pixel within the feature map based on their spatial positions. As depicted in Figure 5, the feature map generated by the MCEM and the up-sampled feature map are respectively subjected to $F_{conv}^{1\times 1}$ convolution operations. After the convolution operations, the feature maps are passed through a batch normalization layer to improve the stability and generalization capability. Subsequently, the above features are connected, and then nonlinear is introduced through ReLU function, which further increases the nonlinearity and expression ability of the network. Moreover, the output is passed through a $F_{conv}^{1\times 1}$ convolution and a batch normalization layer to extract higher-level feature information to a certain extent. Finally, pixel-level prediction and segmentation are performed through Sigmoid function to obtain the final output feature map.

## 3 Experimental results

**FIGURE 6:**
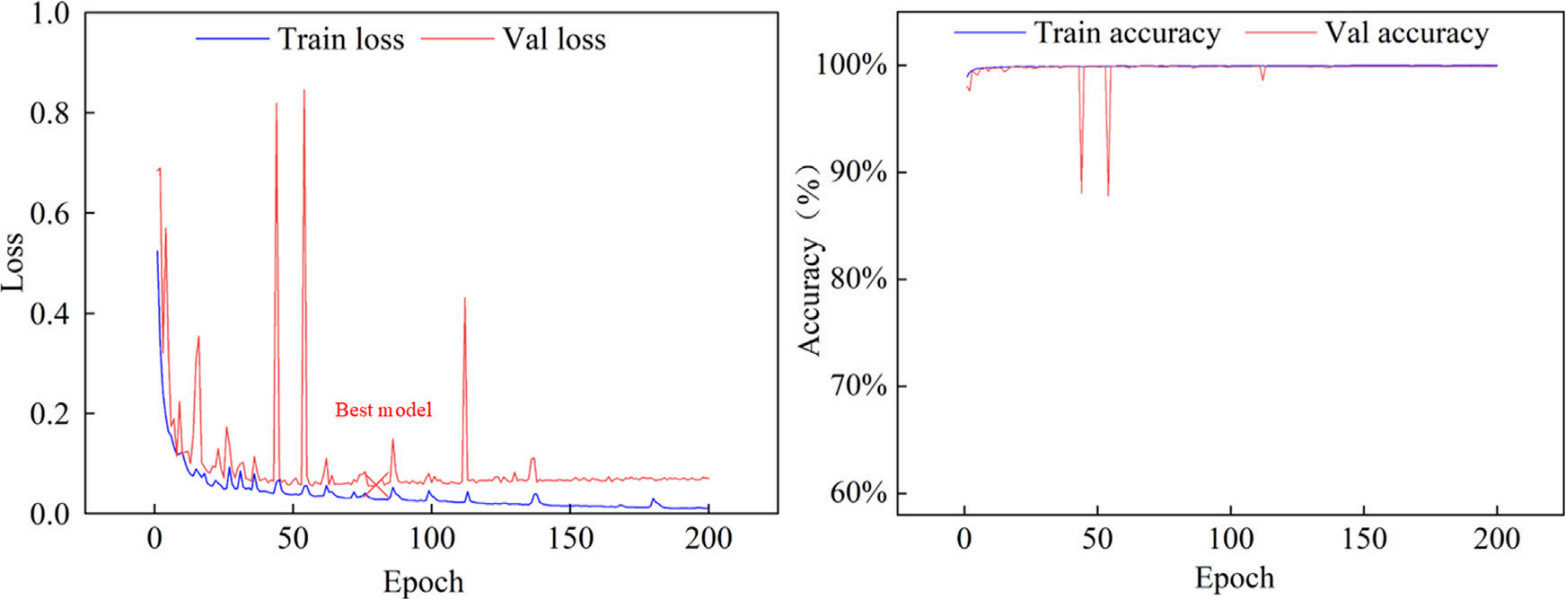
Iterative parameter training curve on the original jaw cyst dataset.

In this section, we present a comprehensive series of experiments aimed at evaluating the performance of the proposed MARes-Net. All experiments were conducted in a Python 3 environment using the powerful computational capabilities of the Quadro RTX 6000 GPU, alongside TensorFlow 2.4.0 as the framework. We set the batch size to 4, which strikes a balance between memory efficiency and training speed. The training process was carried out over 200 epochs, the learning rate to 0.001, and we employed the Adam optimizer to optimize the training process and Dice as the loss function. As shown in Table 1, the image data comes from the records of Quzhou People’s Hospital. The jaw cyst dataset consisted of 1535 images, of which 306 were used for testing, 922 for training, and 307 for validation. The dataset after data augmentation consisted of 4,602 data sets, 910 for testing, 2,765 for training, and 920 for validation. These experimental settings ensure the consistency of the experiments and provide us with reliable evaluation results. After the model is built and trained, we monitor its performance over the training period to ensure it is learning effectively and improving its predictive capabilities. Figure 6 illustrates the changes in both loss and accuracy values throughout the training process on the original jaw cyst dataset.

**TABLE 1:** The parameter about the datasets.

Dataset	Training data	Validation data	Test data	Image size	Label size
Original jaw cyst dataset	922	307	306	256 × 256	256 × 256
Dataset after data augmentation	2765	920	917	256 × 256	256 × 256

To assess the performance of MARes-Net, we use the following metrics: precision (Shu et al., 2024; Xia et al., 2024), recall (Sun et al., 2024; Yuan et al., 2024), IoU (Fan et al., 2024; Wang et al., 2024), and F1-score (Li et al., 2024; Tang et al., 2024). IoU is commonly used to measure the performance of object detection or segmentation tasks, evaluating the similarity between the segmentation mask predicted by the model and the ground-truth mask. Precision measures the proportion of correctly predicted positives over those predicted by the model, and recall measures the ability of the model to identify positives, which is the probability of correctly predicting all positives. The F1-score combines precision and recall through its harmonic mean, providing a balanced performance metric. Using these metrics, we can comprehensively evaluate the model’s performance in segmentation tasks and its effectiveness in handling unbalanced data sets. Their relevant formulas are as follows:

$$precision=\frac{TP}{TP+FP}$$ (6)

$$recall=\frac{TP}{TP+FN}$$ (7)

$$IoU=\frac{TP}{TP+FP+FN}$$ (8)

$$F1-score=\frac{2\times precision\times recall}{precision+recall}$$ (9)

The learning rate is an important parameter in neural network training. It controls the step size that the model takes along the gradient direction each time the parameters are updated. A learning rate that is too high can cause the model to converge too quickly to a suboptimal solution or even diverge, as the updates might overshoot the optimal point. On the other hand, a learning rate that is too low can make the training process excessively slow, potentially getting stuck in local minima or requiring an impractical amount of time to converge. Therefore, selecting an appropriate learning rate is vital, as it has a direct impact on both the convergence speed and the final performance of the model. To understand the effect of different learning rates on our model’s performance, we conducted a series of experiments on the original jaw cyst dataset with various learning rate values, as summarized in Table 2. The experimental data clearly show that setting the learning rate to 0.001 results in optimal performance.

**TABLE 2:** The effect of different learning rates on the original jaw cyst dataset.

Learning rate	Precision (%)	Recall (%)	IoU (%)	F1-score (%)
0.01	92.68	91.16	83.56	91.28
0.0025	94.09	91.73	83.96	90.92
0.005	92.29	91.49	82.91	91.76
0.0001	93.23	91.49	83.61	90.65
0.001	93.84	93.70	86.17	93.21

### 3.1 Ablation experiment

#### 3.1.1 The role of residual connection

In addition, we also tested whether there is a residual connection. Table 3 presents the performance metrics of MARes-Net on the original jaw cyst dataset, including precision, recall, IoU and F1-score. Specifically, MARes-Net achieved precision of 93.70%, recall of 93.83%, IoU of 86.17%, and F1-score of 93.21%. These results clearly indicate that the inclusion of residual connections substantially enhances the model’s performance across all key metrics.

**TABLE 3:** Residual connection experiment.

	Precision (%)	Recall (%)	IoU (%)	F1-score (%)
No residual connection	92.56	93.73	84.35	91.96
With residual connections	93.70	93.83	86.17	93.21

**FIGURE 7:**
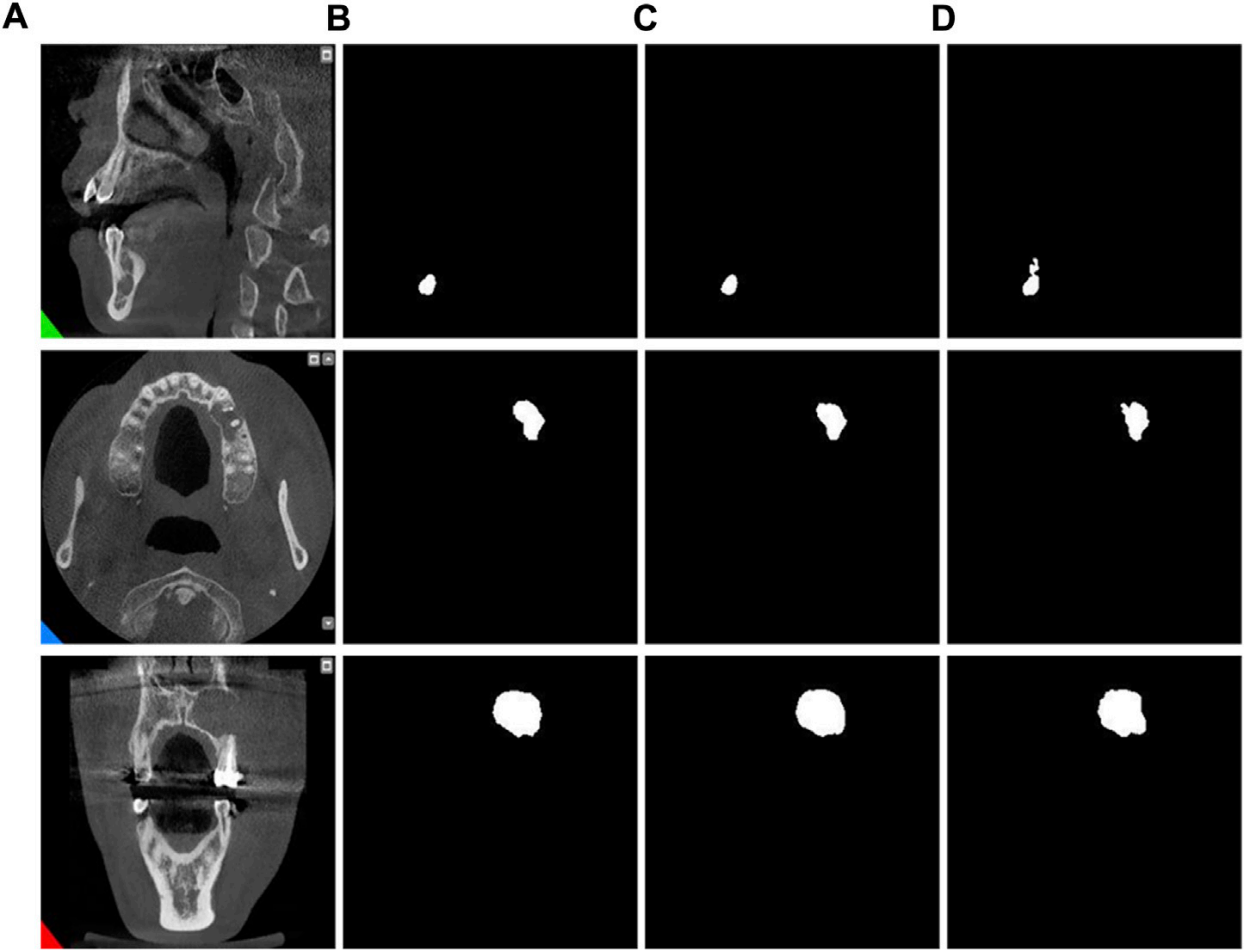
Residual connection experiment. **(A)** Original images. **(B)** Label images. **(C)** No residual connection. **(D)** With residual connection.

By observing the predicted segmentation shown in Figure 7, we can find that in a network without residual connections, the segmentation map has defects in feature extraction and cannot perfectly extract all features. In contrast, networks with residual connections can better overcome the problem of feature extraction and generate more accurate segmentation maps. This shows the importance of the residual connection structure in the MARes-Net, which can help the network better learn and transfer feature information. By ensuring that crucial features are preserved and effectively utilized throughout the network, residual connections significantly contribute to the model’s ability to generate high-quality, accurate segmentation results.

#### 3.1.2 Different attention modules

In this section, we adopt CBAM in the scale-aware feature extraction module to obtain channel and spatial information. To assess the effectiveness of CBAM, we conducted comparative experiments on the original jaw cyst dataset with different connection methods: direct connection, CAM connection, and SAM connection. As listed in Table 4, we can observe that the network connected using CBAM is slightly inferior to several other connection methods in the precision indicator, but is significantly better than the other several connection methods in the recall indicator. By comparing the results in Table 4, we can conclude that the network with CBAM connections performs better in terms of overall performance.

**TABLE 4:** The Impact of different attention modules.

	Precision (%)	Recall (%)	IoU (%)	F1-score (%)
Direct connection	94.07	92.09	84.50	90.92
CAM connection	93.69	92.83	84.93	92.25
SAM connection	94.34	91.89	84.30	91.05
CBAM connection	93.84	93.70	86.17	93.21

**FIGURE 8:**
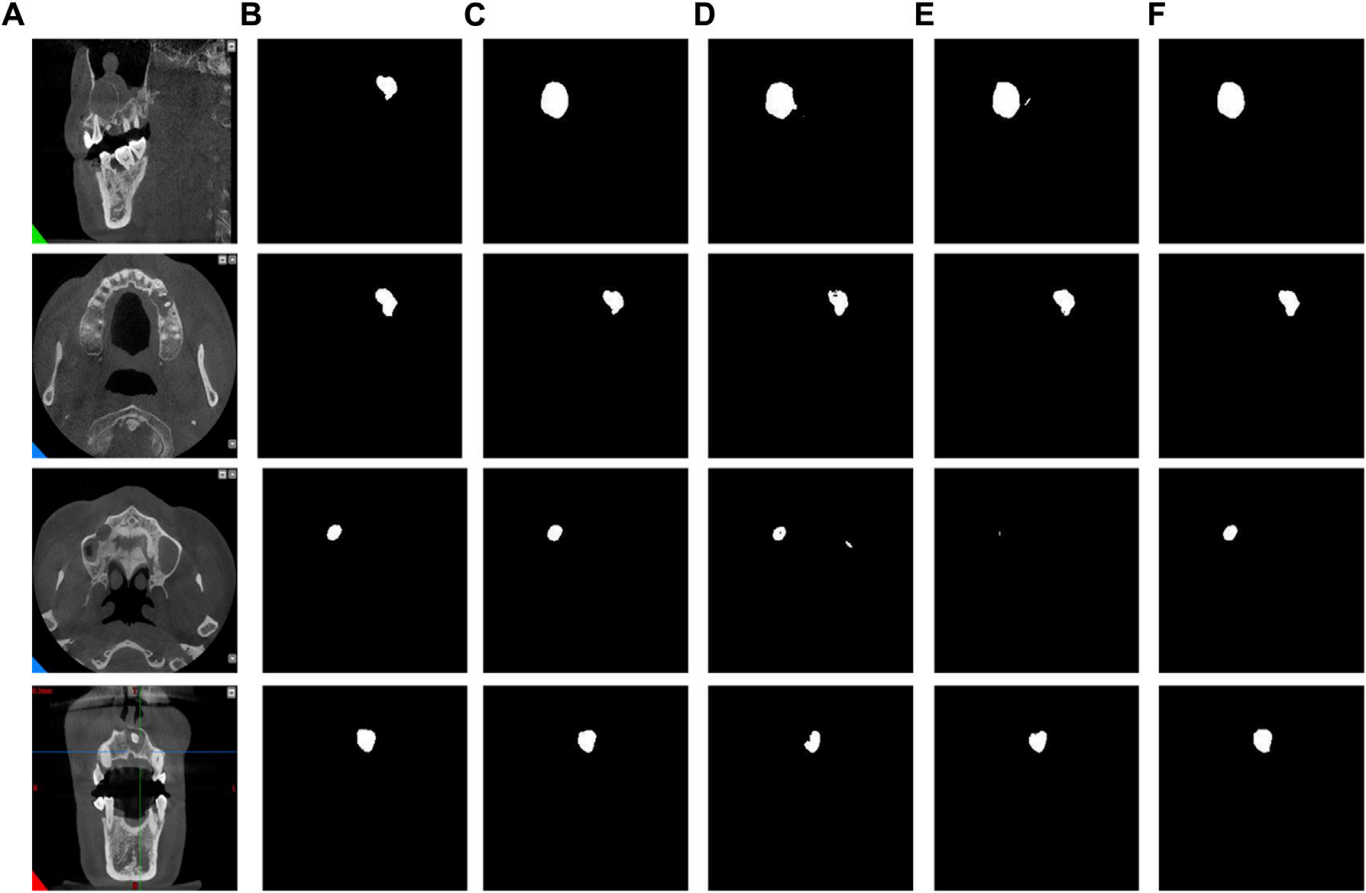
The results of different attention modules. **(A)** Original images. **(B)** Label images. **(C)** Direct connection. **(D)** CAM connection. **(E)** SAM connection. **(F)** CBAM connection.

In addition, through the results of image segmentation Figure 8, we can see that when using SAM and CAM connections alone, there is an erroneous segmentation of the area of interest and the image cannot be perfectly segmented. However, when using CBAM connections, we can observe that the accuracy of segmented regions is improved, indicating that CBAM connections can better guide the network to accurately segment regions of interest.

#### 3.1.3 Other module experiments

**FIGURE 9:**
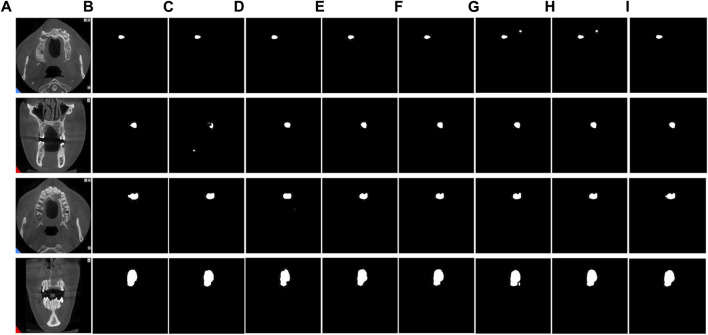
Results of ablation experiments for each module. **(A)** Original images. **(B)** Label images. **(C)** ResU + AG. **(D)** ResU + SFEM. **(E)** ResU + MCEM. **(F)** ResU + AG + SFEM. **(G)** ResU + AG + MCEM. **(H)** ResU + SFEM + MCEM. **(I)** ResU + AG + SFEM + MCEM.

To evaluate the superiority of MARes-Net, we conducted other module ablation experiments on the original jaw cyst dataset. These experiments were designed to understand the contribution of each component within the network. Table 5 presents a detailed comparison of the performance metrics with various configurations of MARes-Net. When there was only a multi-scale compression excitation module, the model had the lowest comprehensive data performance, such as precision of 93.07%, recall of 92.02%, IoU of 82.02% and F1-score of 90.19%. Conversely, when all modules were integrated into the MARes-Net network structure, the model demonstrated substantial improvements across all evaluation metrics and both indicators maintained high values: precision reached 93.84%, recall was at 93.70%, and the comprehensive performance metrics were equally impressive with an F1-score of 93.21%, and an IoU of 86.17%. In addition, through Figure 9, we can observe that the partially combined network structure performs poorly in the segmentation task of jaw cysts and cannot accurately capture image information. In contrast, the complete MARes-Net network shows more accurate capabilities in image segmentation and localization.

**TABLE 5:** Ablation experiments of each module.

	Precision (%)	Recall (%)	IoU (%)	F1-score (%)
ResU + AG	93.07	92.41	83.32	91.92
ResU + SFEM	93.62	92.65	82.25	92.16
ResU + MCEM	93.62	92.02	82.02	90.19
ResU + AG + SFEM	94.16	91.38	83.65	91.59
ResU + AG + MCEM	91.72	93.26	83.71	92.74
ResU + SFEM + MCEM	93.22	91.70	83.97	91.35
ResU + AG + SFEM + MCEM	93.84	93.70	86.17	93.21

### 3.2 Compare networks

**FIGURE 10:**
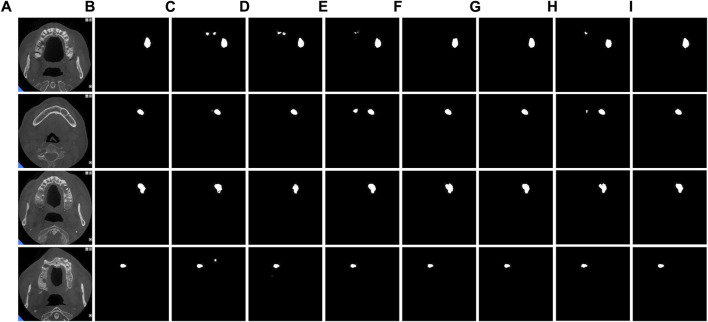
Comparison with other models on the original jaw cyst dataset. **(A)** Original image. **(B)** Label image. **(C–I)** are the results of HRNet, ICNet, scSEUnet, SK_U_Net, CLNet, CLCI_Net, U-Net, MARes-Net.

To comprehensively evaluate the accuracy of MARes-Net in the task of original jaw cyst image segmentation, we compared it with seven classic and recently released models, including HRNet (Sun et al., 2019), ICNet (Zhao et al., 2018), scSEUnet (Roy et al., 2018), SK_U_Net (Byra et al., 2020), CLNet (Zheng et al., 2021), CLCI_Net (Yang et al., 2019), U-Net (Ronneberger et al., 2015). The experimental data are shown in Table 6 below. The results show that MARes-Net is slightly inferior to scSEUnet in terms of precision, but performs best on other evaluation indicators. Compared with U-Net, MARes-Net improved IoU by 4.36%, and F1-score by 2.46%. Due to the variety and complexity of images of jaw cysts, identifying the cyst area presents certain challenges. It is obvious from Figure 10 in the third row that the compared networks failed to fully capture information in segmentation, resulting in missing information and incomplete segmentation results. In contrast, our proposed MARes-Net has obvious advantages in segmentation and localization.

**TABLE 6:** Comparison of our model with other models on the original jaw cyst dataset.

	Precision (%)	Recall (%)	IoU (%)	F1-score (%)
HRNet (Sun et al., 2019)	93.33	90.46	82.34	90.06
ICNet (Zhao et al., 2018)	91.41	87.24	79.47	88.08
scSEUnet (Roy et al., 2018)	94.02	91.93	78.69	87.82
SK_U_Net (Byra et al., 2020)	93.86	92.85	82.91	90.44
CLNet (Zheng et al., 2021)	92.85	92.04	80.34	88.67
CLCI_Net (Yang et al., 2019)	93.37	91.28	82.48	90.02
U-Net (Ronneberger et al., 2015)	93.38	91.41	81.81	89.55
MARes-Net	93.84	93.70	86.17	92.47

**FIGURE 11:**
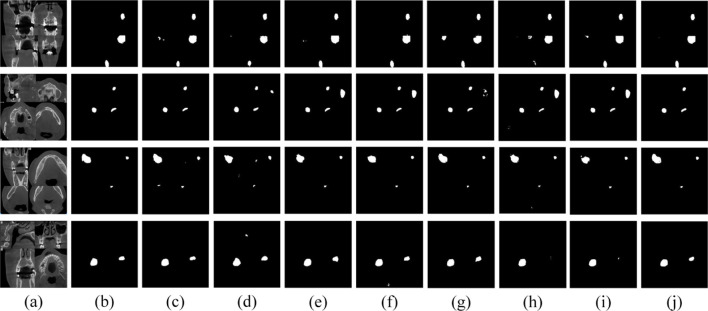
Visual results of various models on data augmentation dataset. **(A)** Original image. **(B)** Label image. **(C–J)** are the results of HRNet, ICNet, scSEUnet, SK_U_Net, CLNet, CLCI_Net, U-Net, MARes-Net.

To address the challenges posed by limited sample sizes and class imbalance in jaw cyst datasets, we employed various data augmentation techniques to enhance the diversity of our training and validation datasets. These techniques included random transformations, scale adjustments, displacement transformations, and local stretching, aimed at improving the model’s comprehensive recognition ability for jaw cysts. Through these strategies, we aimed to enhance the model’s robustness and generalization across different conditions, ensuring accurate identification of jaw cysts from various angles and scenarios. During the experimental phase, we trained and evaluated these strategies on augmented datasets using seven classical and recently published models (HRNet (Sun et al., 2019), ICNet (Zhao et al., 2018), scSEUnet (Roy et al., 2018), SK_U_Net (Byra et al., 2020), CLNet (Zheng et al., 2021), CLCI_Net (Yang et al., 2019), U-Net (Ronneberger et al., 2015). Detailed results are presented in Table 7. The findings indicated a slight decrease in IoU and F1 scores for these models on the augmented dataset. However, surprisingly, MARes-Net, in combination with SFEM and MCEM, achieved improved segmentation results when handling complex images. As illustrated in Figure 11, MARes-Net demonstrated its capability to produce high-quality and accurate segmentation results from these lesion segmentation images.

**TABLE 7:** Results of our method with other models on data augmentation dataset.

	Precision (%)	Recall (%)	IoU (%)	F1-score (%)
HRNet Sun et al. (2019)	94.34	83.97	80.40	88.87
ICNet Zhao et al. (2018)	90.16	76.86	70.40	81.68
scSEUnet Roy et al. (2018)	95.00	87.01	78.11	91.78
SK_U_Net Byra et al. (2020)	95.50	85.83	82.30	91.03
CLNet Zheng et al. (2021)	85.30	84.22	78.98	89.44
CLCI_Net Yang et al. (2019)	59.81	73.47	43.57	79.06
U-Net Ronneberger et al. (2015)	95.27	94.60	81.51	89.67
MARes-Net	95.24	85.25	82.15	90.25

### 3.3 Computational analysis and efficiency comparison

In model evaluation, the number of parameters and training time is a critical aspect. The number of parameters directly impacts the training duration of the model. Generally, a higher parameter count indicates greater model complexity, necessitating more computational resources and time to complete training. However, models with more parameters also pose significant challenges in terms of training time and resource consumption, requiring extended training periods, large datasets, and high-performance computing resources. As shown in Table 8, ICNet (Zhao et al., 2018), scSEUnet (Roy et al., 2018), and UNet (Ronneberger et al., 2015) have notably fewer parameters, albeit at the expense of performance. Table 8 illustrates that these three models exhibit significantly lower IoU and F1 scores compared to other models in the comparison. Conversely, despite MARes-Net requiring more time and parameters during training, its integration of SFEM and MCEM enhances the incorporation of contextual information, thereby improving its ability to capture complex data patterns. This advantage translates into superior predictive accuracy.

**TABLE 8:** Comparison of parameter counts and computational time among different models.

	Parameter (M)	Time (ms/step)
HRNet Sun et al. (2019)	7.98	350
ICNet Zhao et al. (2018)	1.71	150
scSEUnet Roy et al. (2018)	1.96	135
SK_U_Net Byra et al. (2020)	3.94	240
CLNet Zheng et al. (2021)	3.60	130
CLCI_Net Yang et al. (2019)	12.56	565
U-Net Ronneberger et al. (2015)	1.97	110
MARes-Net	4.58	200

## 4 Conclusion

As deep learning continues to achieve remarkable success in the field of medical image segmentation, this paper focuses on the image segmentation of jaw cysts and proposes an innovative network structure, namely, MARes-Net. Firstly, the overall integration of residual connections helps model optimization and training. Secondly, the scale-aware feature extraction module is used to combine atrous convolution and CBAM mechanisms to increase the receptive field and accurately locate the area of interest. Furthermore, the feature map is stimulated and compressed through a multi-scale compression excitation module to obtain rich contextual information and improve the model’s performance capabilities. Finally, an attention gate module is introduced to adjust the feature map obtained by the multi-scale compression excitation module and up-sampling to improve the model’s attention to the target area and reduce interference to the background area. A series of experimental results on the original jaw cyst dataset show that the precision, recall, IoU, and F1-score, and of our proposed method can reach 93.84%, 93.70%, 86.17%, and 93.21%, respectively., which is significantly better than other classic models cited in this article. This research aims to apply innovative technologies to a wider range of medical image analysis tasks and bring important breakthroughs and contributions to the research and application of medical image segmentation.

While our approach has been remarkably successful in segmenting jaw cyst images, the complexity of the model presents challenges in achieving real-time performance and computational efficiency. Future research should give priority to improving the real-time and computational efficiency of the model to better adapt to clinical applications. This requires not only optimizing existing models, but also exploring and applying new deep learning techniques to further improve the accuracy and reliability of medical image segmentation. These advances will allow us to better meet actual medical needs and provide patients with more effective diagnostic and treatment support.

## Data Availability

The original contributions presented in the study are included in the article/supplementary material, further inquiries can be directed to the corresponding authors.
